# Quality of primary palliative care for older people with mild and severe dementia: an international mortality follow-back study using quality indicators

**DOI:** 10.1093/ageing/afy087

**Published:** 2018-06-08

**Authors:** Rose Miranda, Yolanda W H Penders, Tinne Smets, Luc Deliens, Guido Miccinesi, Tomás Vega Alonso, Sarah Moreels, Lieve Van den Block

**Affiliations:** 1 End-of-Life Care Research Group, Vrije Universiteit Brussel (VUB) & Ghent University, Brussels, Belgium; 2 Department of Family Medicine and Chronic Care, Vrije Universiteit Brussel (VUB), Brussels, Belgium; 3 Department of Medical Oncology, Ghent University Hospital, Ghent, Belgium; 4 Clinical and Descriptive Epidemiology Unit, Cancer Prevention and Research Institute, Florence, Italy; 5 Public Health Directorate, Regional Ministry of Health (Dirección General de Salud Pública, Consellería de Sanidad), Castile and Leon, Valladolid, Spain; 6 Scientific Institute of Public Health (Wetenschappelijk Instituut Volksgezondheid, Institut Scientifique de Santé Publique), Unit of Health Services Research, Brussels, Belgium

**Keywords:** palliative care, primary palliative care, general practice, dementia, quality indicators, older people

## Abstract

**Background:**

measuring the quality of primary palliative care for older people with dementia in different countries is important to identify areas where improvements can be made.

**Objective:**

using quality indicators (QIs), we systematically investigated the overall quality of primary palliative care for older people with dementia in three different countries.

**Design/setting:**

a mortality follow-back survey through nation- and region-wide representative Sentinel Networks of General Practitioners (GPs) in Belgium, Italy and Spain. GPs registered all patient deaths in their practice. We applied a set of nine QIs developed through literature review and expert consensus.

**Subjects:**

patients aged 65 or older, who died non-suddenly with mild or severe dementia as judged by GPs (*n* = 874).

**Results:**

findings showed significantly different QI scores between Belgium and Italy for regular pain measurement (mild dementia: BE = 44%, IT = 12%, SP = 50% | severe dementia: BE = 41%, IT = 9%, SP = 47%), acceptance of approaching death (mild: BE = 59%, IT = 48%, SP = 33% | severe: BE = 41%, IT = 21%, SP = 20%), patient–GP communication about illness (mild: BE = 42%, IT = 6%, SP = 20%) and involvement of specialised palliative services (mild: BE = 60%, IT = 20%, SP = 77%). The scores in Belgium differed from Italy and Spain for patient–GP communication about medical treatments (mild: BE = 34%, IT = 12%, SP = 4%) and repeated multidisciplinary consultations (mild: BE = 39%, IT = 5%, SP = 8% | severe: BE = 36%, IT = 10%, SP = 8%). The scores for relative-GP communication, patient death outside hospitals and bereavement counselling did not differ between countries.

**Conclusion:**

while the countries studied differed considerably in the overall quality of primary palliative care, they have similarities in room for improvement, in particular, pain measurement and prevention of avoidable hospitalisations.

## Introduction

Dementia affects about 47 million people worldwide and is projected to almost triple by 2050 as populations age [1]. It is characterised by widespread physical, cognitive and behavioural impairment which may lead to severe disabilities that persist until death [2, 3]. To improve the quality of life of older people with dementia and their families, a recent white paper from the European Association for Palliative Care recommends palliative care [4]. However, concerns have been raised about the poor quality and access to palliative care for older people with dementia [1], particularly in primary care where the majority will be cared for [5].

Existing studies using death certificates show that home death is rare among people with dementia and the majority die in nursing homes [6, 7]. Those dying in nursing homes are more likely to receive burdensome interventions, be hospitalised unnecessarily and die with great suffering [8–10]. In primary care, evidence on some circumstances of those dying with mild or severe dementia in Belgium, Italy and Spain suggests that although transfer rates are low and treatment aims are aligned with palliative care recommendations, access to specialised palliative services and communication with general practitioners (GPs) remains poor [11, 12]. However, previous studies no longer reflect the latest evidence in primary care, due partly to recent changes in palliative care legal frameworks and funding arrangements in these countries [13, 14]. Additionally, they included people with dementia who died ‘suddenly’ [11, 12], who may not have been recognised as being in the final stage of life and thus in need of palliative care [15]. Finally, they neither showed a comprehensive overview of the most important aspects of palliative care nor systematically measured its quality, something that could be achieved by using a core set of quality indicators (QIs) [16–18].

QIs are explicitly defined and measurable items referring to the structure, process or outcome of care, which can be used to capture the quality of care on an aggregated, for example, national level [16–18]. Because there is still no norm to determine when a certain QI score indicates ‘high-quality’ primary palliative care, comparing QI scores between countries is useful. Such cross-country comparisons can shed light on the average level of quality across different countries [19], giving insight into where improvements can be made [20].

In this study, we aimed to answer the research question: ‘What is the quality of primary palliative care in Belgium, Italy and Spain for older people who died non-suddenly with mild or severe dementia?’. We applied a core set of nine validated QIs, which cover eight important domains of palliative care and are highly applicable and easily implementable in primary care in an international context according to experts. We used international population-based data (2013–15) from existing representative GP Sentinel Networks in Belgium, Italy and Spain. Comparing these countries is interesting, as they have all integrated palliative care in their health systems, but their health systems are different and the outcomes may still vary [13, 14, 21].

## Methods

### Design

The current study is part of the European Sentinel Network Monitoring End-of-Life Care (EUROSENTIMELC), a mortality follow-back study monitoring palliative care in population-based samples of death in Belgium, Italy and Spain. Data were collected through existing Sentinel Networks of GPs, an epidemiological surveillance system that voluntarily monitors health problems in primary care. The network in Belgium is representative for age, sex and the geographical distribution of GPs in the country, while the network in Spain covers the Castile and Leon and the Valencian regions. In Italy, we used a national representative GP network that only performed end-of-life care registrations. Using a standardised registration form, GPs registered deaths weekly among patients in their practice aged 18 or older from January 2013 to December 2014 in Belgium and Spain and from June 2013 to May 2015 in Italy. In completing the registration form, GPs may also use medical files. The design and aims of EUROSENTIMELC have been explained in detail elsewhere [22].

### Sample

Data were collected on 2,435 patients in Belgium, 983 in Italy and 591 in Spain. All those aged 65 or older who died non-suddenly with either mild or severe dementia as judged by the GP were included, making a total sample of 874 (531 in Belgium, 242 in Italy and 101 in Spain).

### Measurements

The registration form consists of structured and closed-ended items surveying information from the GPs about QIs and patient characteristics. Based on their knowledge and expertise, the GPs estimated whether the patient had dementia (‘yes, mild dementia’, ‘yes, severe dementia’, ‘no’ and ‘unknown’ (considered as ‘no’)) and whether they died suddenly.

#### Dependent variables—selecting QIs

Table 1 summarises the selection of EUROSENTIMELC QIs and the calculation of QI scores. The core set of EUROSENTIMELC QIs was based on previous work of Leemans *et al. *[18], who identified nine important domains of palliative care (i.e. physical, psychosocial, communication with patients and relatives, multidisciplinary consultation, type of end-of-life care, continuity of care, support for relatives and structure of care) and evaluated a set of QIs designed to measure the quality of palliative care services in Belgium. Of those QIs found to have good face validity, feasibility, discriminative power and usefulness, we identified 43 QIs that can be measured retrospectively using GPs as respondents. These QIs were reformulated as questions, so that sentinel GPs could respond to them. Based on usefulness and relevance to primary palliative care in an international context, 22 primary palliative care experts from Belgium, Italy and Spain, the Netherlands and France evaluated the 43 QIs on a scale of 1–10. Those with a minimum average score of 7.5 remained. Where possible, we reduced the QIs to 1–2 per domain by selecting the best scored QIs. We only selected the QIs measuring the process and outcomes of care, resulting in 17 QIs covering eight palliative care domains, which were included in the registration form.

**Table 1.** The selection of EUROSENTIMELC QIs and the calculation of QI scores

**Phase 1**	**From previous work** [18]**, we identified 43 validated palliative care QIs covering nine domains of palliative care, which can be measured retrospectively using GPs and respondents**		
**Phase 2**	**Expert consultation and steering group to evaluate the 43 QIs and reduce the QI set to 1**–**2 per domain—Result:** 17 QIs included in the registration form.		
**Multistep process**	**Further evaluation of the psychometric qualities of the QIs based on data quality and usability—** We only selected the QIs covering the process and outcomes of care domains and excluded the ‘structure of care domain’. **Result:** Nine QIs covering eight domains of palliative care were selected and operationalised (see the list below). Eight QIs were discarded: (i) number of contacts between the GP and the patient in the last 3 months of life, (ii) percentage of patients whose GP is aware of their wishes regarding resuscitation, (iii) percentage of patients whose nominated proxy decision-maker was involved when the patient became mentally incompetent, (iv) percentage of patients with more than one visit to an emergency department in the last 30 days before their death, (v) percentage of patients who are admitted to an intensive care department in the last 30 days before their death, (vi) percentage of patients with more than one hospital admissions in last 30 days, (vii) percentage of patients who *remained* in their preferred location in the last month before their death and (viii) percentage of patients who *died* in their preferred location		
**Palliative care domains**		**Numerator and denominator^a^**	**Calculation of QI scores (questions, answers and scoring)**
**1: Physical aspects of care**		**(QI 1) Numerator:** Number of patients whose pain was measured often or very often in the last 3 months of life	**Question:** How often did you or another caregiver measure the patient’s pain (with or without using a pain scale) in the last 3 months of life? **| Answer:** never; rarely; occasionally; often; very often **- Positive score:** If the GP knew pain to be measured ‘often’ or ‘very often’
**2: Psychosocial and spiritual aspects of care**		**(QI 2) Numerator:** Number of patients who accepted death completely or for the most part** Denominator:** Number of patients for whom the GP answered anything but ‘I don’t know’	**Question:** According to you, did the patient accept his/her approaching end? **| Answer:** Yes, completely; yes, for the most part; no, not entirely; no, not all; I don’t know (reported separately in Table 1). **- Positive score:** If the GP thought the patient had accepted their nearing end. All ‘don’t know’ answers were not included in this indicator (*n* was reported)
**3: Information, communication, planning and decision-making with the patient**		**(QI 3.1) Numerator:** Number of patients with whom the GP discussed at least three of the listed topics	**Question:** Put a cross against topics you have discussed with the patient **Answer:** Diagnosis; course of the disease/prognosis; the approaching end of life; advantages and disadvantages of the treatments; options in terms of end-of-life care **- Positive score:** If the GP communicated about at least three of the five illness-related topics
		**(QI 3.2) Numerator:** Number of patients who expressed a specific wish about a medical treatment (i.e. ‘Yes’)	**Question:** Did the patient ever express specific wishes about a medical treatment that he/she would or would not want in the final phase of life? **| Answer:** Yes; No; Don’t know **- Positive score:** Yes | The answers ‘don’t know’ were considered as ‘no’, as this QI focuses on GP-patient discussion
**4: Information, communication, planning and decision-making with family and friends**		**(QI 4) Numerator:** Number of patients for whom the GP discussed at least three of the listed topics with a relative	**Question:** Put a cross against topics you have discussed with the relative **Answer:** Diagnosis; course of the disease/prognosis; the approaching end of life; advantages and disadvantages of the treatments; options in terms of end-of-life care **- Positive score:** If the GP communicated about at least three of the five illness-related topics
**5: Information, communication, planning and decision-making with other care providers**		**(QI 5) Numerator:** Number of patients for whom a multidisciplinary consultation took place approximately once a week or approximately everyday	**Question:** How often in the last month of life did a pre-planned multidisciplinary consultation take place (face-to-face or via phone) between the care providers to discuss the care objectives and/or options in terms of palliative care? **Answer:** No such consultation/once in the last month of life/approximately once a week/approximately everyday **- Positive score:** If the multidisciplinary consultation occurred ‘once a week’ or ‘once a day or more’
**6: Type of palliative care**		**(QI 6) Numerator:** Number of patients for whom at least one specialised palliative care services [19] was initiated in the last 3 months of life	**Question:** Which specialised palliative care initiatives were involved in the last 3 months of this patient’s life? **Answer:** Country-specific specialised palliative care initiatives; other, namely…; none **- Positive score:** If specialised palliative care services were involved in the last 3 months of life
**7: Coordination and continuity of care**		**(QI 7) Numerator:** Number of patients who did not die in hospital (exclusive palliative care unit)	**Question:** Place of death? **| Answer:** At home or living with family (incl. service flat); care home: home for elderly persons/nursing home; hospital (excl. palliative care unit); palliative care unit (hospital); elsewhere, please specify **- Positive score:** If death occurred outside the hospital. The original indicator was ‘percentage of people who died at home’. We adapted it to allow for deaths in palliative care units, which are indicative of high-quality palliative care
**8: Support for family/friends and informal carers**		**(QI 8) Numerator:** Number of patients for whom the GP has contacted or plans to contact the relatives with regard to bereavement counselling	**Question:** After the death, did you have contact with any of the relatives with regard to bereavement counselling? **Answer:** Yes, once; Yes, more than once; No, but has been planned; No and not planning to **- Positive score:** If GP has contacted or plans to contact the relatives with regard to bereavement counselling

^a^Denominator: all patients for whom this question was answered (unless otherwise indicated).

Through a multistep process of assessing the psychometric qualities of the QIs explained hereunder, we finally selected nine and discarded eight QIs (e.g. ‘Percentage of patients with more than one visit to an emergency department in the last 30 days before their death’ due to 26% missing cases) [23]. For the detailed selection of QIs, see Appendix 1 in the Supplementary Data are available in *Age and Ageing* online.

- All questions were analysed for data quality. Any QIs with missing values of 10% or more were excluded, assuming that GPs may have had difficulty answering.

- All questions were also analysed based on usability by examining potential ceiling or floor effects and variability between disease groups and countries. Questions with positive answers of more than 90% or less than 10% over all countries were excluded.
- The question and answer categories were used to calculate the following core set of QIs, of which two cover the third palliative care domain:
  (1) Percentage of patients whose pain was known by the GP to be monitored regularly during the last 3 months of life
  (2) Percentage of patients known by the GP to have accepted that they were nearing the end of their life
  (3.1) and (4) Extent to which patients and relatives receive information from the GP about diagnosis, prognosis, disease progression, advantages and disadvantages of treatments and palliative care options
  (3.2) Percentage of patients who expressed a specific wish about a medical treatment
  (5) Repeated (on several occasions) formal multidisciplinary consultation with and between care providers (between settings, including GP) about care goals and palliative care option
  (6) Percentage who received palliative care services [24] involved in last 3 months of life
  (7) Percentage of patients who did not die in a regular hospital unit
  (8) Percentage of patients for whom the GP has contacted or has plans to contact the relatives regarding bereavement counselling

#### Independent variables

Besides age at death and gender, GPs indicated the cause of death: ‘malignancy’, ‘cardiovascular disease (excluding stroke)’, ‘disease of the nervous system’, ‘respiratory diseases’ and ‘other (specified)’. The place of longest residence in the last year of life was also requested: ‘at home or living with family’, ‘care home’ and ‘elsewhere’.

### Data analyses

Generalised linear mixed model (GLMM) analysis was carried out to calculate cross-country differences in characteristics of those with mild and severe dementia, while accounting for their clustering within GP practices. GLMM analysis was also conducted to analyse differences in QI scores between countries, while adjusting for sample characteristics that varied between countries and accounting for the clustering. Statistical analyses were conducted with IBM SPSS Statistics 24: Release 24 (IBM Corporation).

### Ethics approval

Ethics approval was obtained from the Ethical Review Board of Brussels University Hospital of the Vrije Universiteit Brussel and from the Local Ethical Committee Comitato Etico della Azienda Sanitaria Firenze in Tuscany (April 2013). No formal ethical approval is required to collect posthumous anonymous patient data in Spain.

## Results

### Patient characteristics

Among the mild dementia group, those in Belgium were the youngest (mean age 86.4 years), and in Spain the oldest (88.9; *P* = 0.026, Table 2); the average age for those with severe dementia was 85.8 in Belgium to 88.5 in Spain (*P* = 0.001). All groups were predominantly female from 53.1% in Spain to 74.6% in Italy (*P* = 0.031). In the last year of life of those with mild dementia, 55.7% resided in care homes in Belgium, whereas 85.1% in Italy and 78.8% in Spain resided at home (*P* < 0.001) as with severe dementia, though the difference was not significant. The most common cause of death in mild dementia in Belgium and Italy was cardiovascular disease (24.2% and 39.6%, respectively), while in Spain, a third died of stroke (*P* = 0.009). In all countries, almost a third of those with severe dementia died from nervous system disease.

**Table 2.** Patient characteristics between Belgium, Italy and Spain (*n* = 874)

	Mild dementia (*n* = 385)				Severe dementia (*n* = 489)			
	Belgium (*n* = 219)	Italy (*n* = 114)	Spain (*n* = 52)	*P-*value^b^	Belgium (*n* = 312)	Italy (*n* = 128)	Spain (*n* = 49)	*P-* value^b^
	*n* (%)	*n* (%)	*n* (%)		*n* (%)	*n* (%)	*n* (%)	
Mean age at death [SD]	86.4 [7.3]	87.6 [5.7]	88.9 [6.2]	0.026^a^	85.8 [6.7]	87.9 [6.7]	88.5 [4.7]	0.001^a^
Gender, female	137 (63.1)	66 (59.5)	36 (69.2)	0.502	216 (69.7)	94 (74.6)	26 (53.1)	0.031^a^
Longest place of residence in the last year of life^c^				<0.001^a^				<0.001^a^
At home	95 (43.4)	97 (85.1)	41 (78.8)		87 (28.0)	104 (81.3)	32 (66.7)	
Care home^d^	122 (55.7)	16 (14.0)	11 (21.2)		223 (71.7)	24 (18.8)	16 (33.3)	
Main cause of death				0.009^a^				0.080
Malignancy	51 (23.3)	9 (8.1)	9 (17.3)		32 (10.3)	5 (4.0)	8 (16.3)	
Cardiovascular disease	53 (24.2)	44 (39.6)	13 (25.0)		59 (19.0)	34 (27.0)	6 (12.2)	
Disease of nervous system	25 (11.4)	8 (7.2)	5 (9.6)		100 (32.2)	39 (31.0)	19 (38.8)	
Respiratory disease	32 (14.6)	18 (16.2)	3 (5.8)		19 (6.1)	15 (11.9)	4 (8.2)	
Stroke (CVA)	23 (16.0)	17 (13.5)	6 (30.8)		43 (13.8)	11 (8.7)	3 (6.1)	
Other	35 (16.0)	15 (13.5)	16 (30.8)		58 (18.6)	22 (17.5)	9 (18.4)	

Missing cases for mild dementia, gender, *n* = 5 (BE = 2 | IT = 3); cause of death, *n* = 3 (IT = 3).

Missing cases for severe dementia, gender, *n* = 4 (BE = 2 | IT = 2); longest place of residence prior to death, *n* = 3 (BE = 2 | SP = 1); cause of death, *n* = 3 (BE = 1 | IT = 2).

SD, standard deviation; CVA, cerebrovascular accident.

^a^Significant at the 0.05 probability level.

^b^*P*-value was determined by conducting multilevel mixed model analysis to account for the clustering at the level of GPs.

^c^Longest place of residence in the last year of life: ‘Elsewhere’ reported as missing cases (mild dementia, *n*: Belgium, 2; Italy, 1; Spain, 0 | severe dementia, *n*: Belgium, 1; Italy, 0; Spain, 0).

^d^Includes care/nursing homes in Belgium and Italy and residential homes in Spain.

### Quality of primary palliative care in Belgium, Italy and Spain

In the last 3 months of life, GPs indicated that regular pain measurement was conducted in 44% of those with mild dementia in Belgium, 12% in Italy (OR = 0.15, 95% CI = 0.06–0.40) and 50% in Spain (n.s., Table 3). This pattern was also found in the severe dementia group (Belgium (41%), Italy (9%; OR = 0.10, 95% CI = 0.04–0.29), Spain (47%, n.s.)). In Belgium, 59% of those who died with mild dementia accepted their death according to the GP, compared with 48% in Italy (OR = 0.26, 95% CI = 0.10–0.65) and 33% in Spain (n.s.) with a similar pattern for severe dementia (Belgium (41%), Italy (21%; OR 0.25, 95% CI 0.08–0.76) and Spain (20%, n.s.)).

**Table 3.** Scores of the nine QIs for patients with mild and severe dementia between Belgium, Italy and Spain (*n* = 874)

	Mild dementia (*n* = 385)					Severe dementia (*n* = 489)				
	Belgium (*n* = 219)	Italy (*n* = 114)		Spain (*n* = 52)		Belgium (*n* = 312)	Italy (*n* = 128)		Spain (*n* = 49)	
	*n* (%)	*n* (%)	OR (95% CI)	*n* (%)	OR (95% CI)	*n* (%)	*n* (%)	OR (95% CI)	*n* (%)	OR (95% CI)
**QI 1.** Pain measured often or very often in last 3 months of life	95 (44)	14 (12)	0.15 (0.06–0.40)^a^	25 (50)	1.67 (0.63–4.46)	124 (41)	11 (9)	0.10 (0.04–0.29)^a^	22 (47)	2.28 (0.76–6.88)
**QI 2.** GP thinks that patient was able to accept their approaching end completely or for the most part^b^	127 (59)	54 (48)	0.26 (0.10–0.65)^a^	17 (33)	0.32 (0.10–1.00)	123 (41)	26 (21)	0.25 (0.08–0.76)^a^	9 (20)	0.26 (0.07–1.01)
**QI 3.1.** GP discussed at least three illness-related topics^c^ with patient	76 (42)	7 (6)	0.09 (0.03–0.22)^a^	3 (20)	0.33 (0.08–1.30)	28 (14)	0 (0)	NA	1 (11)	0.93 (0.10–8.62)
**QI 3.2.** GP was aware of patient preferences about medical treatments	74 (34)	13 (12)	0.21 (0.09–0.47)^a^	2 (4)	0.07 (0.01–0.30)^a^	31 (10)	4 (3)	0.40 (0.15–1.12)	2 (4)	0.52 (0.14–2.03)
**QI 4.** GP discussed at least three illness-related topics^c^ with relatives	162 (81)	93 (82)	0.61 (0.27–1.38)	39 (85)	0.99 (0.35–2.81)	244 (84)	107 (84)	0.95 (0.42–2.13)	42 (88)	1.03 (0.34–3.14)
**Qi 5.** Multidisciplinary consultation at least once a week during the last month of life	84 (39)	5 (5)	0.08 (0.03–0.24)^a^	4 (8)	0.11 (0.04–0.37)^a^	111 (36)	13 (10)	0.30 (0.15–0.61)^a^	4 (8)	0.21 (0.08–0.55)^a^
**QI 6.** Palliative care services involved in last three months of life	125 (60)	20 (20)	0.17 (0.08–0.38)^a^	30 (77)	2.94 (1.14–7.61)	185 (62)	12 (11)	0.08 (0.03–0.18)^a^	29 (73)	1.83 (0.72–4.67)
**QI 7.** Patient did not die in hospital^d^	162 (74)	83 (73)	1.59 (0.86–2.93)	32 (64)	0.82 (0.39–1.69)	264 (85)	105 (82)	1.68 (0.86–3.28)	34 (72)	0.61 (0.27–1.39)
**QI 8.** GP contacted or plans to contact relatives about bereavement counselling	150 (69)	72 (65)	0.64 (0.28–1.44)	32 (62)	0.55 (0.23–1.33)	189 (62)	84 (67)	0.88 (0.44–1.73)	27 (56)	0.49 (0.22–1.09)

**Missing cases for patients with mild dementia**, QI 1, *n* = 4 (BE = 1 | IT = 1 | SP = 2); QI 2, *n* = 4 (BE = 2 | IT = 1 | SP = 1); QI 3.1, *n* = 76 (BE = 39 | SP = 37); QI 3.2, *n* = 1 (IT = 1); QI 4, *n* = 25 (BE = 19 | SP = 6); QI 5, *n* = 7 (BE = 2 | IT = 2 | SP = 3); QI 6, *n* = 36 (BE = 11 | IT = 13 | SP = 12); QI 7, *n* = 2 (SP = 2); QI 8, *n* = 5 (BE = 2 | IT = 3).

**Missing cases for patients with severe dementia**, QI 1, *n* = 14 (BE = 10 | IT = 2 | SP = 2); QI 2, *n* = 13 (BE = 8 | IT = 2 | SP = 3); QI 3.1, *n* = 155 (BE = 115 | SP = 40); QI 3.2, *n* = 4 (BE = 2 | IT = 2); QI 4, *n* = 23 (BE = 22 | SP = 1); QI 5, *n* = 11 (BE = 7 | IT = 3 | SP = 1); QI 6, *n* = 34 (BE = 12 | IT = 14 | SP = 8); QI 7, *n* = 4 (BE = 2 | SP = 2); QI 8, *n* = 9 (BE = 6 | IT = 2 | SP = 1).

Reference group = Belgium. Accounted for the clustering at the level of GPs and adjusted for age, gender, cause of death, and longest place of residence in the last year of life.

CI, confidence interval; OR, odds ratio; Ref., reference category; NA, not applicable.

^a^Significant at the 0.05 probability level.

^b^Excluded ‘don’t know’ (mild dementia, 132; severe dementia, 282).

^c^The topics are diagnosis, course of the disease/prognosis, the approaching end of life, advantages and disadvantages of the treatments, options in terms of end-of-life care.

^d^Regular hospital wards excluding palliative care units.

GPs indicated that at least three of the five illness-related topics were discussed with patients with mild dementia more often in Belgium (42%) than in Italy (6%; OR = 0.09, 95%CI = 0.03–0.22), though no significant difference was found between Belgium and Spain (20%, n.s.). Additionally, the preferences of patients with mild dementia about end-of-life treatment were discussed more frequently in Belgium (34%) than in Italy (12%; OR = 0.21, 95%CI = 0.09–0.47) and Spain (4%; OR = 0.07, 95%CI = 0.01–0.30); in severe dementia, figures were lower (0–14%) but no significant difference was found between countries, nor with communication between GPs and relatives (81–85% mild dementia; 84–88% severe dementia).

In the last month of life, repeated multidisciplinary consultations about end-of-life care for those with mild dementia were more likely in Belgium (39%) than in Italy (5%; OR = 0.08, 95% CI = 0.03–0.24) and Spain (8%; OR 0.11, 95 %CI 0.04–0.37); (severe dementia: Belgium (36%), Italy (10%; OR = 0.30, 95 %CI = 0.15–0.61) and Spain (8%; OR = 0.21, 95% CI = 0.08–0.55)). During the last 3 months of life, specialised palliative services were involved more frequently in mild dementia in Belgium (60%) than in Italy (20%; OR = 0.17, 95% CI = 0.08–0.38), while no difference was found in Spain (77%); (severe dementia Belgium (62%), Italy (11%; OR 0.08, 95%CI = 0.03) and Spain (73%, n.s)).

The percentages of those who did not die in a hospital varied from 64–74% (mild dementia) and 72–85% (severe dementia), though there was no cross-country difference. The percentages of relatives of patients, who were contacted or are planned to be contacted by GPs about bereavement counselling were similar across countries (62–69% for mild dementia and 56–67% for severe dementia). A visual overview of the QIs in Belgium, Italy and Spain is shown in three radar charts in Appendix 2 in the Supplementary Data are available in *Age and Ageing* online.

## Discussion

Our findings show considerable cross-country differences on regular pain measurement, acceptance of approaching death, patient–GP communication about illness and medical treatments, involvement of specialised palliative services and repeated multidisciplinary consultations about end-of-life care. QI scores in Belgium were higher than Italy, but not appreciably higher than Spain. Scores for relative-GP communication, death outside hospital and bereavement counselling for relatives did not differ between countries and ranged from 56% to 88%.

Our study was the first to measure the quality of primary palliative care for older people with mild or severe dementia using a core set of validated QIs, which cover eight important palliative care domains and are highly applicable and easily implementable for this study according to experts from five different countries. We also provided a good understanding of the final phase of life from a population-based perspective. Through the GP Sentinel Networks, we had representative samples of patient deaths in primary care [22] and included people who had and had not received specialised palliative services. The inclusion of all non-sudden deaths in our study also enabled us to assess the quality of care delivered in the context of dying.

Nevertheless, our findings should be interpreted in light of the study’s limitations. First, we relied on the GP's estimation of the presence and severity of dementia. GP’s specificity in diagnosing dementia is good, making false positive less likely to occur [25]. However, there might be limited misclassifications, which could explain the difference in proportions of dementia severity. Second, to limit recall bias, we instructed GPs to use medical files and register deaths within a week.

Although Belgium, Italy and Spain have national palliative care legal frameworks and have integrated it into their health systems [13, 14], our findings suggest that the overall quality in Belgium is higher than Italy, but not appreciably higher than in Spain. This may be because Belgium has the highest ratio of palliative care resources per million inhabitants [13, 14] and provides detailed guidelines for palliative home care teams and networks, promoting collaborative practice and reciprocal sharing of knowledge and expertise with GPs [26, 27]. This collaborative practice may also explain our findings regarding the significantly higher scores for repeated multidisciplinary end-of-life care consultations in Belgium. The comparable QI scores for Spain and Belgium may result from their efforts to expand palliative care from cancer to non-cancer patients, including older people and those with dementia [13, 28]. Whereas in Italy, palliative care remains focused on the needs of cancer patients [13], which may explain why Italy had the lowest scores in five of the six QIs wherein the countries studied differed significantly.

While the countries studied differ in the overall quality of primary palliative care, they have similarities in room for improvement. First, the pain of more than half of patients across countries was not regularly measured, which is comparable to what was found in long-term and acute care settings [29]. Pain is highly prevalent among older people with dementia, and if not treated adequately may lead to depression, agitation and aggression [30, 31]. Even where self-reporting is not possible due to cognitive decline, other strategies can be used, such as direct observation of behavioural cues and the use of validated tools such as Pain Assessment in Advanced Dementia Scale [32, 33]. In line with an earlier study [12], more than two-thirds of patients, particularly in Italy and Spain, appeared to have poor communication with GPs. The relatively higher score for patient–GP communication in Belgium may be due to their continued efforts in advance care planning [23] and the culture of wanting to be informed about health-related issues [34]. While this poor communication with patient may be understandable due to cognitive decline, our study suggests that this is a problem even for people with mild dementia. Similar with an earlier study [12], we found high levels of relative-GP communication across the three countries, implying that GPs communicate more often with relatives than with patients, which seems to be an alternative to the poor communication with patients. Finally, although most older people prefer to die at home or in a care home [35], about a third of people studied died in hospital. Reducing avoidable hospitalisations at the end-of-life may prevent unnecessarily burdensome medical treatments and lower risk for functional decline and mortality [10].

## Conclusion

Our study suggests considerable cross-country differences and similarities in the overall quality of primary palliative care for older people with dementia, potentially as a result of different national health systems (e.g. palliative care resources and focus on dementia) and culture. It also highlights similar opportunities for improvement, in particular, pain measurement and prevention of avoidable hospitalisations. Our findings are useful to guide efforts to improve primary palliative care for older people with dementia, while the core set of QIs is useful for monitoring the overall quality of care over time.

**Key points**

- Many older people with dementia particularly in primary care receive poor quality and access to palliative care.
- To identify room for improvement, we assessed the quality of primary palliative care in dementia in Belgium, Italy and Spain.
- Quality was systematically assessed using a set of QIs developed through literature review and expert consensus.
- The countries differed in quality, but they have similar opportunities for improvement, e.g. pain measurement and hospitalisation.
- Our findings are useful to reflect on how primary palliative care can be improved for older people with dementia.

## Supplementary Material

Supplementary Data
