# Supplementary material for: Quality of primary palliative care for older people with mild and severe dementia: an international mortality follow-back study using quality indicators
Source: Age Ageing. 2018 Jun 8;47(6):824–33. doi: 10.1093/ageing/afy087 (PMC6201823; doi:10.1093/ageing/afy087)
Supplement: Supplementary Data [file afy087_aa-18-0021-file002.doc]

Appendix 1. List of selected and discarded quality indicators

|  | **Palliative care domains and quality indicators** | **Mean score for usefulness and relevance†** | **Selection after the multi-step process ^‡^** |
| --- | --- | --- | --- |
|  | **Domain 1: Physical aspects of care** |  |  |
| **D1.1** | Percentage of patients whose pain was known by the GP to be monitored regularly during the last three months of life. | 7,5 (selected) | Selected (QI 1) |
| **D1.2** | Percentage of patients who were known by the GP to be monitored regularly for delirium during the last three months of life. | 6,0 |  |
| **D1.3** | Percentage of patients whose symptoms were under control during the last week of life. | 7,5 | Not included in the questionnaire |
|  | **Domain 2: Psychological, social and spiritual treatment and care** |  |  |
| **D2.1** | Percentage of patients who were known by the GP to be receiving regular assessment for anxiety during the last three months of the patient's life. | 6,5 |  |
| **D2.2** | Percentage of patients who were known by the GP to be monitored regularly for psychosocial problems during the last three months of the patient's life. | 7,3 |  |
| **D2.3** | Percentage of patients who were known by the GP to be monitored regularly for religious/spiritual/existential problems during the last three months of the patient's life. | 6,0 |  |
| **D2.4** | Percentage of patients known by the GP to have accepted that they were nearing the end of their life. | 8,0 (selected) | Selected (QI 2) |
|  | **Domain 3: Information, communication, planning and decision-making with the patient** |  |  |
| **D3.1** | Extent to which patients receive information from the GP about diagnosis, prognosis, disease progression, advantages and disadvantages of treatments, palliative care options. | 8,0 (selected) | Selected (QI 3.1) |
| **D3.2** | Extent to which care providers indicate that the patient has been asked how he/she feels about end-of-life decisions. | 6,0 |  |
| **D3.3** | Number of contacts between the GP and the patient in the last 3 months of life. | 7,5 (selected) | Discarded, due to lack of clarity on causal relationship |
| **D3.4** | Percentage of patients whose wishes regarding care goals have been discussed with the GP. | 8,0 (selected | Selected (QI 3.2) |
| **D3.5** | Percentage of patients whose GP is aware of their wishes regarding resuscitation. | 7,5 (selected) | Discarded, due to overlap with the QI for ‘wishes for treatment’ |
| **D3.6** | Percentage of patients whose nominated proxy decision-maker was involved when the patient became mentally incompetent. | 7,8 (selected) | Discarded, due to low quality (50% missing cases) and low usability (ceiling effect) |
| **D3.7** | Percentage of patients for whom documentation is available regarding the end-of-life care and treatment that they wish to receive. | 7,0 |  |
| **D3.8** | When a patient expressed specific wishes regarding a medical treatment that he/she does or does not want to receive towards the end of life then no further medical procedures or treatments are performed in the last week of life that are not in accordance with the wishes expressed. | 7,3 |  |
|  | **Domain 4 Information, communication, planning and decision-making with the family and friends** |  |  |
| **D4.1** | Extent to which care providers keep the family and friends informed about the patient’s condition and treatments. | 6,5 |  |
| **D4.2** | Extent to which the family and friends are informed that the patient is nearing the end of his/her life. | 7,0 (selected) | Selected (QI 4) |
| **D4.3** | Extent to which family and friends were consulted if decisions have been made about tube feeding or artificial fluids and feeding. | 6,3 |  |
|  | **Domain 5: Information, communication, planning and decision-making with other care providers** |  |  |
| **D5.1** | Repeated (on several occasions) formal multidisciplinary consultation with and between care providers (between settings, including GP) about the patient’s care goals and palliative care options. | 8,0 (selected | Selected (QI 5) |
|  | **Domain 6: Type of end-of-life care** |  |  |
| **D6.1** | Percentage of patients with an acceptable time span between a new course of chemotherapy and death (according to Grunfeld, more than 2 weeks prior to death) | 6,3 |  |
| **D6.2** | Percentage of patients for whom palliative care is started in good time. | 7,5 (selected) | Selected (QI 6) |
| **D6.3** | Percentage of patients whose GP indicates that the patient has died peacefully. ¶ | Discarded | Discarded |
| **D6.4** | Extent to which patients have the opportunity to be alone with close family and friends *in the last week of life.* | 6,8 |  |
| **D6.5** | Percentage of patients for whom fluids and feeding (not including precautionary intravenous/IV line) were not started in the last month before their death. | 6,3 |  |
|  | **Domain 7: Coordination and continuity of care** |  |  |
| **D7.1** | Extent to which information regarding care and treatment is communicated within the setting/to the next setting prior to a transfer/discharge/admission. | 7,0 |  |
| **D7.2** | Extent to which the care goals and resuscitation status are communicated to the team at the facility to which the patient is transferred. | 7,0 |  |
| **D7.3** | Extent to which information is noted in the medical records: the prognosis, psychosocial symptoms, functional status and symptom burden. | 8,0 | Not included in the questionnaire |
| **D7.4** | Percentage of patients with more than one visit to an emergency department in the last 30 days before their death. | 8,0 (selected) | Discarded, low quality (26% missing cases) |
| **D7.5** | Percentage of patients who are admitted to an intensive care department in the last 30 days before their death. | 7,5 (selected) | Discarded, kept the QI with the highest mean score |
| **D7.6** | Percentage of patients with more than one hospital admissions in last 30 days (according to “Earle/Peruselli”, more than one) | 8,0 (selected) | Discarded, low quality (50% missing cases in Spain) and low usability (ceiling effect) |
| **D7.7** | Percentage of patients who *remained* in their preferred location in the last month before their death*.* | 7,5 (selected) | Discarded, kept the QI with the highest mean score |
| **D7.8** | Percentage of patients who *died* in their preferred location. | 8,0 (selected) | Discarded, low usability (invalid question) |
| **D7.9** | Percentage of patients who die at home. | 7,5 (selected) | Selected (QI 7) |
| **D7.10** | Percentage of patients who know who the designated contact for care is according to the GP. | 6,5 |  |
|  | **Domain 8: Support for family/friends and informal carers** |  |  |
| **D8.1** | Extent to which a regular check is kept on the resilience of the informal carers. | 6,8 |  |
| **D8.2** | Extent to which family and friends get sufficient help with the coordination of practical problems. | 6,5 |  |
| **D8.3** | Extent to which the family and friends get sufficient information about social services and arrangements and about financial allowances. | 6,5 |  |
| **D8.4** | Extent to which close family and friends are advised of the possibilities of aftercare. | 6,5 |  |
| **D8.5** | Extent to which family/friends and informal carers get support with organizing the care process. | 6,5 |  |
| **D8.6** | Follow-up bereavement counselling offered by the physician. | 8,0 (selected) | Selected (QI 8) |
|  | **Domain 9: Structure of care §** |  |  |
| **D9.1** | Percentage of GPs with training in palliative care§ | 8,0 (selected) | Discarded, this domain was excluded |
| **D9.2** | Percentage of GPs who work with a mobile palliative home-care team for x% of their palliative patients (percentage to be determined) § | 8,0 (selected) | Discarded, this domain was excluded |
| **D9.3** | Availability of specialist palliative care at home or in a hospice if the GP should have questions when counseling patients§ | 8,0 (selected) | Discarded, this domain was excluded |
| GP, General Practitioner; QI, quality indicator.  † Results of the expert consultation and steering group  ‡ The remaining quality indicators were further evaluated for data quality and usability, while ensuring to keep at least 1-2 quality indicator per domain  § Collected through yearly registration of GP Sentinel networks  ¶ Quality indicator D6.3 was discarded due to the lack clarity regarding the domain into where it should be categorised (Psychosocial aspect of care rather than type of end-of-life care) | | | |


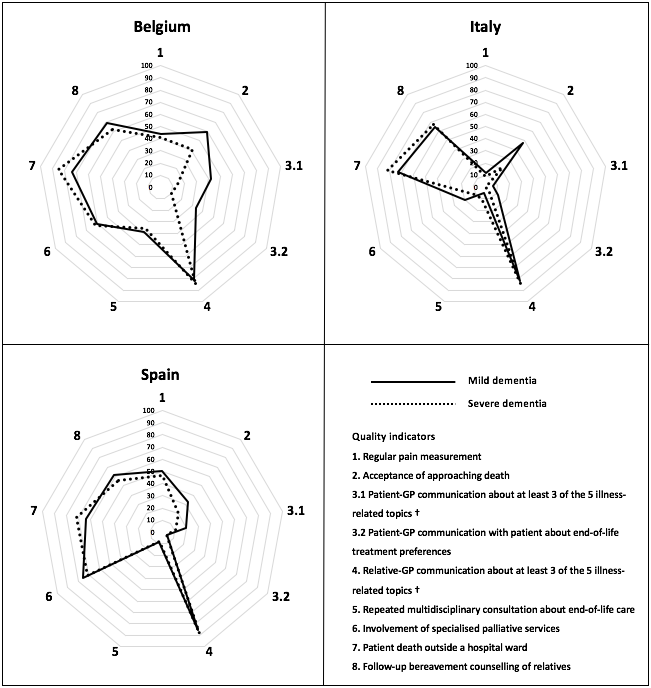
**Appendix 2. Overview of the nine quality indicator scores in Belgium, Italy, and Spain.**

† Illness-related topics included diagnosis, course of the disease/prognosis, the approaching end of life, advantages and disadvantages of

the treatments, and options in terms of end-of-life care
